# The Sea Urchin *Arbacia lixula*: A Novel Natural Source of Astaxanthin

**DOI:** 10.3390/md15060187

**Published:** 2017-06-21

**Authors:** Paola Cirino, Christophe Brunet, Martina Ciaravolo, Christian Galasso, Luigi Musco, Tomás Vega Fernández, Clementina Sansone, Alfonso Toscano

**Affiliations:** 1 Stazione Zoologica Anton Dohrn, Villa Comunale, 80121 Naples, Italy; christophe.brunet@szn.it (C.B.); martinaciaravolo@gmail.com (M.C.); christian.galasso@szn.it (C.G.); luigi.musco@szn.it (L.M.); tomas.vegafernandez@szn.it (T.V.F.); alfonso.toscano@szn.it (A.T.); 2 Department of Veterinary Medicine and Animal Production, University of Naples “Federico II”, Via Federico Delpino 1, 80137 Naples, Italy; 3 National Research Council (CNR)—Institute of Coastal Marine Environment (IAMC), Calata Porta di Massa, 80133 Naples, Italy

**Keywords:** echinoderms, carotenoids, bioactive compounds, DPPH assay, harvesting, dietary supplement

## Abstract

Several echinoderms, including sea urchins, are valuable sources of bioactive compounds but their nutraceutical potential is largely unexplored. In fact, the gonads of some sea urchin species contain antioxidants including carotenoids and polyhydroxylated naphthoquinones (PHNQ’s), such as echinochrome A. Astaxanthin is known to have particular bioactivity for the prevention of neurodegenerative diseases. This carotenoid is produced by microalgae, while several marine invertebrates can bioaccumulate or synthetize it from metabolic precursors. We determined the carotenoid content and analyzed the bioactivity potential of non-harvested Atlantic-Mediterranean sea urchin *Arbacia lixula.* The comparison of methanol crude extracts obtained from eggs of farmed and wild specimens revealed a higher bioactivity in farmed individuals fed with a customized fodder. HPLC-analysis revealed a high concentration of astaxanthin (27.0 μg/mg), which was the only pigment observed. This study highlights the potential of farmed *A. lixula* as a new source of the active stereoisomer of astaxanthin.

## 1. Introduction

Carotenoids are naturally occurring pigments widely distributed among photosynthetic organisms, bacteria, and fungi. Some of the main biological activities and biochemical processes of carotenoids in organisms are pro-vitamin A activity, photoprotection, radical quenching, and immunological modulation [1,2,3]. Animals can obtain carotenoids directly from the diet or through biochemical conversion from dietary precursors. In particular, high carotenoid concentrations occasionally found in the reproductive organs of animals suggest that they could have a role in the reproductive process [3,4]. Carotenes (i.e., β-Carotene) and xanthophylls (i.e., β-echinenone, astaxanthin, lutein, zeaxanthin, and fucoxanthin) are known for their antioxidant activity and for their potential in the prevention and treatment of various diseases. There is increasing evidence that sea urchins, together with other echinoderms, may represent potential sources of valuable bioactive compounds [5,6,7,8,9,10,11]. However, their biotechnological potential remains largely unexplored, and research in this area is still a niche topic [12], particularly in Mediterranean species. Studies conducted on the gonads of some species of sea urchin revealed powerful antioxidant activity due to the presence of carotenoids and polyhydroxylated naphthoquinones (PHNQs), such as echinochrome A [13]. β-Caroten-4-one was first isolated from the gonads of *Paracentrotus lividus* [14], and was named echinenone [15]. β-Echinenone and α-echinenone (α-caroten-4-one) represent the major carotenoids in the gonads of many species of edible sea urchins [3]. The composition and content of these valuable components can differ among different sea urchin species, probably due to the influence of their natural diet as well as physiological processes, i.e., variation in the reproductive phase [16,17]. There is some evidence demonstrating the influence of dietary carotenoids on the color of sea urchin eggs and the potential of sea urchin diets, using both natural and synthetic fodder, in order to assess which is the most effective carotenoid for the desirable colors in several edible species [18,19,20,21,22,23,24].

One of the most active carotenoids that improves egg color is the red ketocarotenoid astaxanthin, which is naturally present in some sea urchin species [3,25]. Astaxanthin is a bioactive carotenoid with potential in the prevention and co-treatment of cancer, chronic inflammatory diseases, metabolic syndrome, diabetes, diabetic nephropathy, cardiovascular diseases, gastrointestinal diseases, liver diseases, neurodegenerative diseases, eye diseases, skin diseases, and exercise-induced fatigue [26]. In particular, astaxanthin is very important in neurodegenerative disease prevention due to its ability to cross the neuroencephalic barrier [27].

Synthetic astaxanthin is produced from petrochemical precursors and consists of a series of stereoisomers distinct from its naturally occurring counterpart. In particular, the synthetic version consists of (3*S*, 3’*S*), (3*R*, 3’*S*), (3*S*, 3’*R*), and (3*R*, 3’*R*), respectively.

Alternatively, astaxanthin may be extracted from the yeast *Phaffia rhodozyma*, and more recently from the bacterium *Lactobacillus plantarum* cultured at a high density. The microalga *Haematococcus pluvialis* is acknowledged as one of the best sources of natural astaxanthin since the maximum amount of the bioactive compound is 38 mg/g of dry matter [28].

The production of astaxanthin from natural sources has become one of the most successful activities in biotechnology, given its great demand in food, feed, nutraceutical and pharmaceutical applications. As a consequence, major efforts to improve astaxanthin production from biological sources instead of synthetic ones are promoted.

Astaxanthin found in marine animals can be the result of accumulation along the food web, in particular starting from phytoplankton that is able to de novo biosynthesize this ketocarotenoid, and/or the secondary product of the metabolic pathway starting from other precursors [29,30].

*A. lixula* is an Atlantic-Mediterranean sea urchin species particularly abundant in the Mediterranean Sea, where it is commonly encountered in shallow rocky bottoms associated with barren habitats [31]. This species is not harvested since it has no commercial value. Moreover, it is considered a thermophilic species that is expanding its range following the general increase in sea surface temperature, especially in the Mediterranean area [32]. Its habitat preference appears to be related to feeding behavior. In fact, the species is considered to be omnivorous since a gut content analysis revealed a prevalence of encrusting calcareous algae in its diet. However, isotopic studies suggest that the main feeding target of *A. lixula* is represented by the sessile fauna associated with calcareous algae, including invertebrates protected by shells [33]. Relatively large amounts of the red pigment echinochrome are present in the *A. lixula* eggs. This colored compound is also responsible for the intense purple color of *A. lixula* eggs. Egg coloration in sea urchins is a result of the deposition of carotenoids obtained from their diet. Both mechanisms—direct deposition of these carotenoids (without modification) and deposition of carotenoid metabolites—have been observed [34,35,36].

Sea urchin eggs are thus a potential source of functional ingredients for nutraceutical and cosmeceutical applications, and *A. lixula* could be a promising species to investigate for industrial exploitation.

The aims of the present study are to (i) assess the presence and the bioactivity of astaxanthin isolated from *A. lixula* eggs; and (ii) test if the *A. lixula* eggs of individuals fed with astaxanthin precursors contain higher concentrations of this active substance than those of wild ones.

## 2. Results

The cultivation protocol has been optimized and astaxanthin-hyperproducing stocks of *A. lixula* can be obtained in a few months. *A. lixula* specimens in culture showed a significantly higher egg production and a prolonged period of reproductive ability compared with wild individuals, allowing the achievement of greater biomass from cultivated sea urchins without seasonal limitation. A substantial amount of biomass was obtained from cultivated sea urchins. A single egg collection from *A. lixula* in culture ranged between 4.5 and 6.9 g, with an average value (±1 SD) of 5.5 ± 0.250. The astaxanthin concentration determined in samples of cultivated *A. lixula* was more than 15 times higher than the concentration in wild samples (see Table 1).

### 2.1. Chemical Analysis

**Figure 1:**
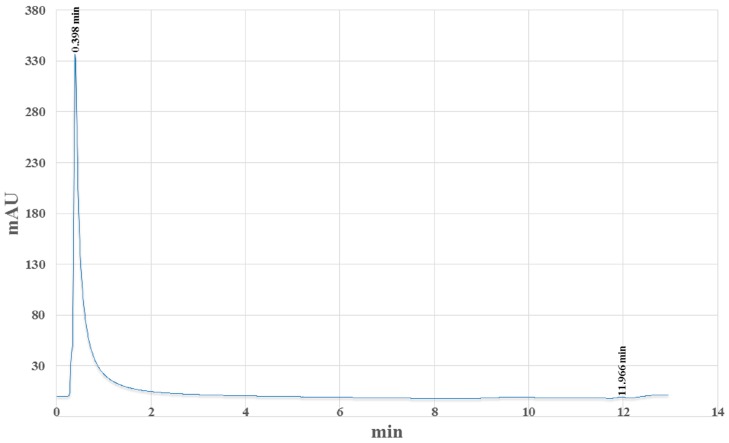
HPLC chromatogram of the *Arbacia lixula* eggs ethanol/water extract. Spectrophotometric detection was setup at 440 nm. The main peak (time: 0.398 min) corresponds to astaxanthin. Axis Y: relative absorbance; Axis X: time (min).

The HPLC chromatogram (Figure 1) revealed that astaxanthin was the main pigment of the extract. The quantity of astaxanthin per dry weight was significantly greater in the eggs of farmed animals than in the eggs of wild ones (*n* = 5; *p* ≤ 0.001; Table 1).

From these results, the amount of astaxanthin production in the eggs of cultivated individuals was estimated (Table 2).

Therefore, we estimated that the total quantity of astaxanthin per dry weight of eggs was about 2.7%.

### 2.2. Radical Scavenging Activity

**Figure 2:**
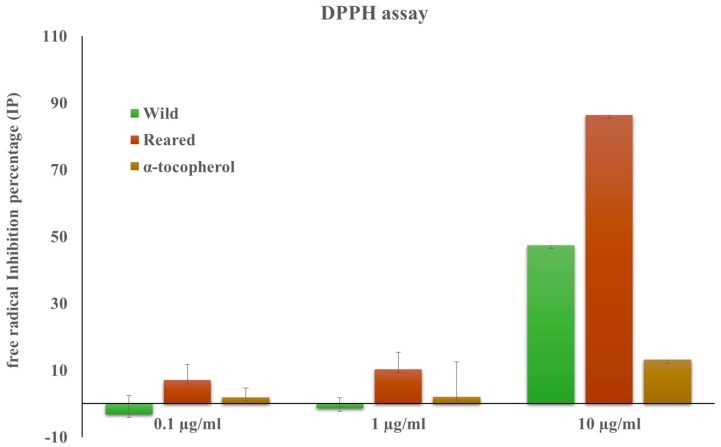
Radical scavenging capacity (Ionization Potential, IP, %) of *Arbacia lixula* eggs aqueous ethanol extract on DPPH free radicals. Values reported are a percentage versus a blank and are represented as the mean ± standard deviation (SD) of five independent samples for each type (wild and farmed).

The aqueous ethanol extract (hereafter referred to as the extract) from *A. lixula* eggs exhibited marked reducing activity toward radical species when the 2,2-diphenyl-1-picrylhydrazyl (DPPH) radical scavenging ability was tested. The addition of extract concentrations of 0.1, 1, and 10 μg/mL taken from each individual resulted in a dose-dependent reduction of the purple radical DPPH into the yellow reduced form for eggs from farmed animals (7.07% ± 5.56% (mean ± SD), 10.31% ± 5.09%, and 86.40% ± 2.82%, respectively). This trend was not consistent across reared and wild individuals due to the lower activity of extracts from wild animals, which were only significantly active (47.44% ± 10.42%) at the highest concentration (Table 3 and Table 4, Figure 2).

## 3. Discussion

The customized fodder enhanced the production and biochemical activity of astaxanthin in farmed *A. lixula* specimens, compared to the wild ones. The increased biochemical activity of astaxanthin extracted from farmed *A. lixula* resulted by testing the aqueous ethanol crude extract of eggs as a DPPH radical scavenger. In fact, we showed that astaxanthin obtained from the eggs of the cultivated *A. lixula* has a potent radical scavenging activity with an inhibition 33% higher than α-tocopherol, used as the benchmark, at a 10 μg/mL concentration.

Carotenoids biosynthesis is biochemically well characterized in the astaxanthin-producing *Hematococcus pluvialis.* In this species, astaxanthin was as the main ketocarotenoid synthesized through biosynthetic reactions starting from the precursor β-Carotene [37,38]. Considering that carotenoids found in animals can be directly accumulated from food and/or partially modified through metabolic reactions [39,40], it is very important to find a way to enhance metabolic pathways in order to optimize the production of bioactive compounds. *Spirulina platensis* has been proved to be a valuable source of carotenoids enhancing the pigmentation in fish [41,42,43,44] and shrimps [45,46], and its inclusion in broodstock diet is recommended to avoid carotenoid deficiency-related problems in shrimp hatcheries [47]. β-Carotene and zeaxanthin, the main carotenoids determined in *S. platensis*, corn, and *Ulva lactuca* [48], are among the reported astaxanthin precursors in animal metabolic pathways [49]. Corn and corn products are considered potential major contributors of dietary zeaxanthin and lutein [50], whereas the green macroalga *U. lactuca* is a good contributor of dietary β-Carotene and other carotenoids [51,52], in addition to important vitamins and minerals. Herein, the optimization of astaxanthin production was obtained through the introduction of intermediates of the biosynthetic pathway of carotenoids, using food as a carrier of target substances. The blue-green algae, *S. platensis*, corn, and *U. lactuca* were used as source of carotenoids due to their content of intermediate products of the astaxanthin biosynthetic pathway.

As previously [48] reported, astaxanthin is contained only in *S. platensis* at the concentration of 0.21 ± 0.02 µg/mL. The feed was administrated twice a week, and the farmed sea urchins were processed after two months of treatment. The total amount of astaxanthin found per gram of eggs was 13.5 mg/g. However, the concentration of bioaccumulated astaxanthin should have been 3.36 μg/g if all the dietary astaxanthin was kept and transferred to the eggs. It is evident that the amount of astaxanthin found in farmed *A. lixula* is higher than the one eventually bioaccumulated, and that it was likely de novo synthesized from the administrated precursors. Further investigations could shed light on the accumulation process and demonstrate the mechanism herein hypothesized.

Comparing the present findings with the results from the available literature [53] it is clear that the eggs of farmed *A. lixula* are a reliable source of astaxanthin, and a convenient alternative to some microalgae. Although the maximum amount of natural astaxanthin obtainable from microalgae up to date is 3.8 mg/g of dry matter (in the case of *H. pluvialis*), the production process can be very expensive due to its chemical purification from other pigments and carotenoids.

## 4. Materials and Methods

### 4.1. Culture System

Sea urchins were hand collected by scuba-diving from rocky shores of the Gulf of Naples, along the southern Tyrrhenian coasts of Italy. Animals were placed in a cooler and carried to the laboratory under moist conditions within 2 h. 

Sea urchins used for the study were held in suspended baskets in a semi-closed recirculating system that receives very low flows of make-up seawater for water losses associated with routine tank cleaning. A centralized Life Support System (LSS) was set to maintain optimal seawater conditions at a constant temperature of 16 ± 1 °C in order to promote the accumulation of gametes and prevent unwanted spawning.

### 4.2. Feeding Practice

We used a mixture of the dry powdered ingredients (animal and vegetable) shaped into Ration Block Food (RBF). We obtained RBF with an average weight of 1 g and feed sea urchins twice a week for two months before the experiments. This quantity corresponded to rations tests. RBFs were always completely eaten. The diet ingredients used were natural and included mussel meal, corn, natural macroalgae (*U. lactuca*) and microalgae (*S. platensis*), fish oil, and mineral supplement. 

### 4.3. Eggs Collection

Gametes were collected by manually pouring from the gonadal pores after animal sacrifice. This method allowed us to gather pure fresh biomass. Only eggs were selected for successive analysis. After collection, they were immediately frozen in liquid nitrogen and stored at −80 °C. The egg biomass collected from single sea urchins ranged between 4.5 and 6.9 g.

### 4.4. Chemical Extraction from Eggs

Extraction was performed according to Sansone et al. [54] and Cutignano et al. [55]. The extraction procedure was conducted under dark conditions, at room temperature, and under nitrogen atmosphere in order to avoid oxidation of the sample. Freeze-dried eggs (~100 mg) were extracted with 1 mL ethanol/water (3/1 *v*/*v*) mixture for 30 min. The mixture was separated by centrifugation at 4500× *g*, for 10 min, at 20 °C, and the supernatant was transferred to a clean tube. The pellet was resuspended in 1 mL of the ethanol/water mixture and extracted for a second time. The aqueous ethanol extract was dried in a rotary vacuum evaporator (Buchi rotavapor R-114). The dry extract was stored under nitrogen atmosphere at −20 °C prior to analysis.

### 4.5. HPLC Analysis

Pigment analysis was conducted by High Performance Liquid Chromatography (HPLC) on an aliquot of the aqueous ethanol extract (10 mg), according to the method described by Brunet et al. [56]. Briefly, the extract was injected in a reversed-phase column (C8 Kinetex column; 50 mm × 4.6 mm; 2.6 µm particle size, Phenomenex^®^, Wilmington, NC, USA) of the Hewlett Packard series 1100 HPLC (Hewlett Packard, Wilmington, NC, USA). Pigments were detected by diode-array spectroscopy (spectrum data collected in the range of 350–750 nm) using a Hewlett Packard photodiode array detector, model DAD series 1100, and absorbance chromatogram was reported at 440 nm. Identification and quantification of pigments were carried out using pigment standards from the D.H.I. Water & Environment (Horsholm, Denmark) [57].

### 4.6. DPPH-Assay

The 2,2-di(4-tert-octylphenyl)-1-picrylhydrazyl (DPPH) was used for the radical scavenging assay (Sigma Aldrich Co., Steinheim, Germany, cat. 257621). Various concentrations (0.1, 1, and 10 µg/mL) of eggs extract were mixed with a final concentration of DPPH of 0.1 mM in methanol, allowed to react for 30 min in the dark, and absorbance was measured at 517 nm using a microplate reader. Results were presented as a percentage of DPPH reduction with respect to the methanol negative control and α-tocopherol used as a positive control.

### 4.7. Experimental Design and Analysis

The study demonstrated two experimental hypotheses derived from the stated aims. The first one (H_11_) predicted antioxidant activity by eggs extract with respect to the statistical control made of methanol. H_11_ was tested at three different doses or concentrations of the eggs extract. The second one (H_12_) predicted higher antioxidant activity by the eggs extract from reared sea urchins with respect to those from wild ones. Both experimental hypotheses were tackled through a Repeated Measures (RM) ANOVA, while the relative intensity of the antioxidant effect was tested across three different doses by an array of a priori comparisons coded as contrast following Neter et al. [58,59]. All *p* values were taken as measures of the relative strength of the evidence against the null hypothesis, H_0_ [59,60]. Individual sea urchins acted as subjects onto which repeated measures (i.e., one extract for each dose level) were taken. Hence, dose of eggs extract (D) was the within-subject or blocking factor, with three levels (low, corresponding to 0.1 μg/mL; medium, 1 μg/mL; and high, 10 μg/mL). Wild versus reared (W vs. R) individuals was the between-subjects factor, with two levels (wild and reared). There were five sea urchin individuals (subjects) within each W vs. R level, for a total of 10 individuals in the experiment. There was one single observation per cell in this design. The response variable was the percentage of antioxidant activity with respect to α-Tocopherol, transformed as *x*’ = *x* + 10 to account for negative values in the DPPH test output. The assumption of homogeneity of variances was ensured via Levene’s test, while that of sphericity was tested through Maucley’s W test.

## 5. Conclusions

Our results demonstrated the double advantage of improving both the available biomass and the concentration of natural astaxanthin in *A. lixula* eggs. The astaxhantin concentration found in the eggs of farmed *A. lixula* was more than 15 times higher than that found in eggs from wild samples. In addition, our approach allows us to base future exploitation of *A. lixula* on cultured stocks without placing any burden on the wild populations. The determination of the pathway leading to the biosynthesis of astaxanthin in this echinoderm exceeds the aims of the present work. However, complete understanding may allow for the further optimization of natural astaxanthin production. Future investigations will allow the transfer of this innovative feeding practice on other sea urchin species to induce the biosynthesis of other bioactive products for nutraceutical and cosmeceutical applications.

## Figures and Tables

**Table 1:** Astaxanthin concentration (mean ± SD) determined in wild and farmed samples of *Arbacia lixula.*

**Wild (*n* = 5)**	**Farmed (*n* = 5)**
1.5 ± 1.8 µg/mg	27.0 ± 7.5 µg/mg

**Table 2:** Total quantity of astaxanthin production in the eggs of cultivated individuals.

**Mean Fresh Weight of Eggs**	**Mean Dry Weight of Eggs**	**Astaxanthin Weight per g of Dry Eggs**
5.5 ± 1.5 g	0.5 ± 0.250 g	0.014 ± 0.004 g

**Table 3:** RM-ANOVA (Repeated Measures-ANalysis of Variance) on transformed (*x*’ = *x* + 10) percentages of DPPH (2,2-diphenyl-1-picrylhydrazyl). SS stands for Sum of Squares error estimator, df indicates the degrees of freedom, MS represents the mean square error estimator, F is the test statistics, p is the probability associated to the observed F, and G-G and H-F refer to Greenhouse-Geisser and Huynh-Feldt corrections for within-subject effects.

Main Effects		SS	df	MS	F	p	G-G e	G-G p	H-F e	H-F p
Wild vs. Reared	WvsR	2313.76	1	2313.76	78.80	2.05 × 10^−5^				
Error		234.91	8	29.36						
Concentration	C	31,520.68	3	10,506.89	430.92	<0.01 × 10^−15^	0.72	1.11 × 10^−15^	1.00	<0.01 × 10^−15^
C × WvsR		2082.90	3	694.30	28.48	4.46 × 10^−8^	0.72	2.46 × 10^−6^	1.00	4.46 × 10^−8^
Error		585.18	24	24.38						

**Table 4:** A priori comparisons across concentration levels within reared (R) and wild (W) *Arbacia lixula* sea urchins. Contrast vectors coded for the unweighted effect of each single concentration level of eggs extract versus the methanol control. C = methanol control, while L, M, and H are low, medium, and high concentrations of the eggs extract, respectively. Data are transformed as *x*’ = *x* + 10 and checked for homogeneity of variances (Levene’s F_1,8_ = 2.53, *p* > 0.1500 for every comparison) and matrix sphericity (Maucley’s W_5_ = 0.49, *p* = 0.4037).

Planned Comparisons within R	SS	df	MS	F	p
L vs. C	120.37	1	120.37	11.92	0.0087
Error	80.81	8	10.10		
M vs. C	259.28	1	259.28	21.55	0.0017
Error	96.27	8	12.03		
H vs. C	18,608.15	1	18,608.15	639.05	6.42 × 10^−9^
Error	232.95	8	29.12		
**Planned Comparisons within W**	**SS**	**df**	**MS**	**F**	**p**
L vs. C	27.84	1	27.84	2.76	0.1355
Error	80.81	8	10.10		
M vs. C	5.01	1	5.01	0.42	0.5367
Error	96.27	8	12.03		
H vs. C	5594.73	1	5594.73	192.14	7.10 × 10^−7^
Error	232.95	8	29.12		
